# Cryptococcal Meningitis Beyond Immunosuppression: A Case Report in an Immunocompetent Individual

**DOI:** 10.1155/crdi/8630525

**Published:** 2026-07-20

**Authors:** Ariba Sultan, Huda Raja, Amna Abrar, Abdullah Nadeem, Shameem Behram Khan, Nahid Raufi

**Affiliations:** ^1^ Department of Medicine, Liaquat National Hospital, Karachi, Pakistan, duhs.edu.pk; ^2^ Dow University of Health Sciences, Karachi, Pakistan, duhs.edu.pk; ^3^ Department of Medicine, Dow University of Health Sciences, Karachi, Pakistan, duhs.edu.pk; ^4^ Department of Medicine, Kabul Medical University, Kabul, Afghanistan, duhs.edu.pk

**Keywords:** cryptococcal meningitis, cryptococcus neoformans, diagnostic challenge, immunocompetent, India ink, tuberculous meningitis

## Abstract

**Background:**

Cryptococcal meningitis is a life‐threatening opportunistic fungal infection predominantly seen in immunocompromised hosts, yet it is increasingly recognized in apparently immunocompetent individuals. In tuberculosis‐endemic regions, its diagnosis is frequently delayed or missed due to overlapping clinical and cerebrospinal fluid findings with tuberculous meningitis.

**Case Presentation:**

A 44‐year‐old immunocompetent woman presented with a two‐month history of progressive diffuse headache and subjective bilateral lower limb weakness. Neurological examination was unremarkable, and initial cerebrospinal fluid analysis revealed marked hypoglycorrhachia (1 mg/dL), elevated protein (356 mg/dL), and lymphocytic pleocytosis. Brain imaging showed leptomeningeal enhancement without mass lesions. Despite negative microbiological testing for tuberculosis, empirical antituberculous therapy was initiated based on presumptive tuberculous meningitis. Pulmonary imaging demonstrated cavitary nodules and calcified lymph nodes. Lack of clinical improvement prompted further investigation; India ink staining and fungal culture of cerebrospinal fluid eventually confirmed *Cryptococcus neoformans* infection. The patient had no evidence of HIV, diabetes, or other immunosuppressive conditions. Induction therapy with liposomal amphotericin B and fluconazole was administered, followed by consolidation and maintenance fluconazole. She showed marked clinical and cerebrospinal fluid improvement and was discharged on oral fluconazole with sustained recovery.

**Discussion:**

This case illustrates the diagnostic challenge of cryptococcal meningitis in immunocompetent patients, particularly in settings where tuberculous meningitis is common. Early reliance on India ink staining and fungal culture is essential when initial tuberculosis workup is negative or when response to antituberculous therapy is poor. The incidental detection of HHV‐6 DNA likely represented viral reactivation without clinical significance.

**Conclusion:**

Cryptococcal meningitis must be considered in immunocompetent patients presenting with chronic meningitis, even in the absence of classic risk factors. Prompt mycological investigation can prevent diagnostic delays and improve outcomes.

## 1. Introduction

*Cryptococcus neoformans* and, less frequently, *Cryptococcus gattii*, are opportunistic fungi most commonly associated with immunocompromised states such as HIV/AIDS and prolonged immunosuppressive therapy [1]. Nonetheless, cryptococcal meningitis (CM) is increasingly reported in apparently immunocompetent patients, with *C. gattii* species complex, particularly *C. gattii and C. deuterogattii,* more often linked to such cases [2, 3]. In countries like Pakistan, CM is usually underdiagnosed due to its overlapping clinical and radiological features with central nervous system tuberculosis [4].

Globally, cryptococcal antigenemia is detected in about 6% of individuals with CD4 counts below 100 cells/μL, representing 278,000 cases annually, with approximately 223,100 progressing to CM. The disease accounts for 181,100 deaths each year and contributes to nearly 15% of AIDS‐related mortality [5]. While mortality is highest in immunocompromised populations, CM remains an uncommon but clinically significant entity in previously healthy individuals [6]. Characteristic prognostic factors include impaired consciousness and elevated cerebrospinal fluid (CSF) opening pressure, both of which are associated with poor outcomes [7–9]. Pathophysiological mechanisms involve blood–brain barrier disruption, impaired glucose transport, and enhanced glycolysis, leading to reduced CSF glucose [10, 11]. Imaging features in immunocompetent patients may show leptomeningeal enhancement, vasculitis, and cystic lesions in Virchow–Robin spaces [12].

Here, we report the case of an immunocompetent female presenting with CM due to *C. neoformans*. The case highlights the diagnostic challenges in tuberculosis‐endemic regions, the characteristic CSF findings, and the therapeutic considerations in resource‐limited settings. This checklist has been completed in accordance with the CAse REport (CARE) guidelines to ensure accurate and transparent reporting of the case (Supporting File 1).

## 2. Case Presentation

A 44‐year‐old previously healthy female, married, housewife by profession, and resident of Quetta with no known comorbidities presented to the emergency department with a 2‐month history of gradual onset, diffuse, intermittent, dull aching headache, progressively worsening, lasting over an hour per episode, mildly relieved with oral paracetamol. Headache was initially associated with vomiting, small quantity, nonprojectile, nonbilious, and non‐bloody, which resolved on its own. She also reported bilateral lower limb pain followed by weakness for the past 15 days. The onset of symptoms was gradual. She denied any history of trauma, seizures, altered level of consciousness, neck stiffness, fever, photophobia, scalp tenderness, increased lacrimation, periorbital pain, or visual disturbances.

On examination, her vital signs were stable: BP 110/70 mmHg, HR 68 bpm, RR 16/min, SpO_2_ 97% on room air, and she was afebrile. Neurologically, she was alert (GCS 15), with intact cranial nerves, normal tone, and no focal neurological deficits. The lower limb weakness was a subjective complaint by the patient (feeling of heaviness and weakness), whereas neurological examination revealed normal muscle power (5/5 in all four limbs), normal tone, and no focal deficit. The complaint likely reflected generalized fatigue or a transient subjective sensation, and it resolved before objective testing. Motor power was normal in all four limbs. Kernig’s and Brudzinski’s signs were negative. Chest examination revealed decreased breath sounds bilaterally (R < L), and the remainder of the systemic examination was unremarkable.

Initial laboratory workup, including CBC, LFTs, renal function, blood glucose, and HIV serology, was unremarkable. MRI brain with contrast revealed leptomeningeal enhancement without evidence of acute infarction or mass effect (Figure 1). Ischemic changes were seen in the right frontal periventricular region and pons. MRA showed moderate segmental narrowing and significantly reduced peripheral cortical branches in both the anterior and posterior circulation. CSF analysis showed a slightly turbid appearance with reduced glucose 1 mg/dL (serum glucose 180 mg/dL), raised protein 356 mg/dL, and WBC count of 140/mm^3^ (70% lymphocytes) (Table 1). Gram stain, AFB smear, GeneXpert and cultures were negative.

**FIGURE 1:**
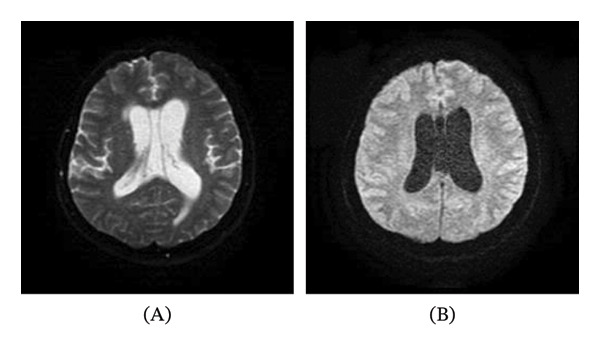
(A) Diffusion‐weighted MRI showing dilated lateral ventricles, periventricular hyperintensity, and compressed cortical sulci. (B) Axial T2/FLAIR MRI demonstrating significant ventriculomegaly, transependymal CSF seepage, and cortical sulcal effacement.

**TABLE 1:** Comparative analysis of CSF findings before and after antifungal therapy.

CSF parameter	Initial analysis	After 2 weeks of therapy
Appearance	Slightly turbid	Clear
Glucose (mg/dL)	1	74
Protein (mg/dL)	356	93
WBC count (/mm^3^)	140	65
Differential count	70% lymphocytes	80% lymphocytes
Fungal culture	Positive	Negative
India ink staining	Positive	Negative

A presumptive diagnosis of tuberculous meningitis was made based on low CSF glucose and lymphocytosis, and empirical antituberculous therapy (ATT) was initiated as per infectious department consult. Despite CSF AFB smear and GeneXpert being negative, ATT was continued. Chest imaging revealed bilateral fibrotic changes and multiple nodular opacities, some with cavitation and calcification (Figure 2). An ultrasound of the chest was done, and samples were sent. A CT chest with contrast was done, which revealed mild fibrotic changes, multiple nodules with cavitation, one in the right upper lobe measured 1.2 × 1.1 cm. Multiple calcified lymph nodes were seen, one measuring 1.7 × 1.8 cm. Cardiomegaly and mildly dilated pulmonary arteries were noticed. The findings were suggestive of fungal infection, and fungal markers were sent. Beta D Glucan levels were raised (80.41), intravenous voriconazole was started, and BAL samples were sent. Repeated CSF analysis showed improved sugars (13), proteins (191) and WBC count (80). CSF viral PCR was positive for Human Herpesvirus 6 and intravenous valacyclovir.

**FIGURE 2:**
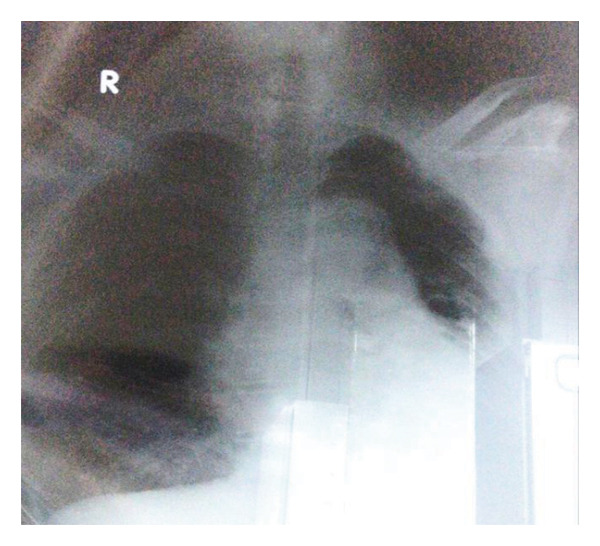
Chest radiograph (posteroanterior view) of the patient demonstrating right‐sided homogenous opacity with blunting of the costophrenic angle, suggestive of pulmonary involvement. Pulmonary cryptococcosis can accompany or precede central nervous system disease even in immunocompetent individuals, indicating disseminated infection.

Despite antiviral treatment, her symptoms persisted. The Infectious Diseases Department advised discontinuing voriconazole. Voriconazole was discontinued, and she was started on intravenous amphotericin B and fluconazole, which were continued for 2 weeks. Repeat CSF was sent for India ink staining and fungal culture, both of which returned positive for *C. neoformans* (Figure 3). Diagnosis of fungal meningitis secondary to *C. neoformans* was made. The patient had no history of corticosteroid use, chemotherapy, solid organ or bone marrow transplantation, malignancy, autoimmune disease, or other immunosuppressive conditions. Repeat HIV serology and HbA1c were negative, ruling out immunocompromise. After two weeks of antifungal therapy, repeat CSF analysis showed clear fluid with normalized glucose (74 mg/dL), reduced protein (93 mg/dL), and a WBC count of 65/mm^3^ (80% lymphocytes) (Table 1). Fungal cultures and CSF cultures showed no growth.

**FIGURE 3:**
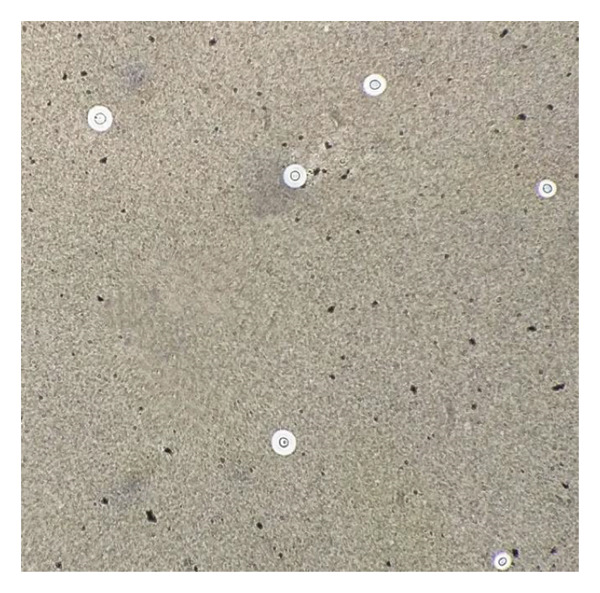
Microscopic image of India ink preparation showing multiple encapsulated round yeast cells of *Cryptococcus neoformans*. The clear halo around the central yeast body represents the characteristic polysaccharide capsule, which is a distinguishing diagnostic feature of cryptococcal infection.

The detection of HHV‐6 DNA in the CSF was interpreted as a possible viral reactivation, often seen in the context of CNS inflammation or as latent chromosomal integration. Given the ongoing headache and lack of improvement, intravenous valacyclovir was initiated empirically. However, the patient’s symptoms persisted, and the subsequent definitive diagnosis of CM, along with a lack of response to antivirals, indicated that HHV‐6 was clinically insignificant. Antiviral therapy was therefore discontinued. The patient’s symptoms, including headache, significantly improved. She was discharged on oral fluconazole 400 mg daily for 8 weeks (consolidation phase), followed by 200 mg daily for 6 months (maintenance phase) (Table 2). Outpatient follow‐up showed continued clinical improvement. Renal function and electrolytes were closely monitored throughout treatment without complications. Clinical timeline from symptom onset to follow‐up is shown in Supporting Table 1. This case report has been prepared in accordance with the CARE guidelines, as outlined in Supporting File 2.

**TABLE 2:** Recommended antifungal treatment phases for cryptococcal meningitis.

Phase	Drug regimen	Duration	Purpose
Induction	Liposomal amphotericin B + fluconazole/flucytosine	2 weeks	Rapid fungal clearance
Consolidation	Fluconazole 400 mg daily	8 weeks	Prevent relapse
Maintenance	Fluconazole 200 mg daily	6–12 months	Reduce recurrence risk

## 3. Discussion

The occurrence of CM in immunocompetent individuals has been associated with rare immune defects or uncommon autoimmune conditions [13, 14]. Reported risk factors include idiopathic CD4+ lymphopenia (present in approximately 27% of cases), pulmonary alveolar proteinosis, monogenic mutations such as GATA2, and polygenic variants including Fcγ Receptor II polymorphisms [13]. In our patient, however, no underlying immune abnormality was identified clinically.

Both *C. neoformans* and *C. gattii* have been isolated in immunocompromised as well as immunocompetent hosts. While *C. neoformans* is more commonly seen in immunocompromised patients, *C. gattii* demonstrates a predilection for those without overt immunodeficiency [15]. In our case, the isolate was confirmed as *C. neoformans*.

Headache and altered mental status represent the most frequent presenting features of CM. Additional manifestations such as fever, nausea, vomiting, and visual disturbances may also occur, the latter often resulting from elevated CSF pressure or direct optic pathway involvement. If left untreated, the disease may progress to seizures, worsening neurological status, or coma [16]. Our patient, however, presented only with headache, and her Glasgow Coma Scale (GCS) remained 15/15 throughout admission.

Despite its relatively low sensitivity (< 86%), India ink staining continues to be widely used for CM diagnosis due to its affordability and accessibility [17]. This method was employed in our case. Nevertheless, when performed as the sole diagnostic tool, up to one in 11 cases may be missed [18].

Typical CSF findings in CM include pleocytosis with mononuclear predominance, elevated protein, and reduced glucose [19]. In our patient, CSF analysis revealed a slightly turbid appearance with glucose markedly reduced to 1 mg/dL (serum glucose 180 mg/dL), protein level of 356 mg/dL, and WBC count of 140/mm^3^ (70% lymphocytes). Following antifungal treatment, repeat CSF analysis demonstrated improvement with normalization of glucose (74 mg/dL), reduction in protein (93 mg/dL), and a decreased WBC count of 65/mm^3^ (80% lymphocytes).

Management of CM involves three therapeutic phases: induction, consolidation, and maintenance [20]. The recommended induction therapy consists of liposomal amphotericin B (L‐AmB) 3–6 mg/kg daily or amphotericin B deoxycholate (D‐AmB) 0.7–1.0 mg/kg daily, combined with flucytosine 100 mg/kg daily (or 75 mg/kg if given intravenously) for 4–6 weeks [21]. In our setting, where flucytosine was not available, the patient received L‐AmB with fluconazole for 2 weeks, consistent with treatment protocols adapted for resource‐limited countries [20].

Our patient’s clinical course aligns closely with the reports of CM in immunocompetent individuals from South Asia. Acharya et al. (2021) described a 48‐year‐old immunocompetent woman with a 2‐month history of headache and low‐grade fever whose CSF demonstrated hypoglycorrhachia and lymphocytic pleocytosis, initially prompting antituberculosis therapy; the correct diagnosis was only established after India ink staining and culture returned positive for *C. neoformans* [3]. Similarly, Arif et al. reported a young immunocompetent man with headache, vomiting, and documented low CSF glucose who was treated empirically for tuberculous meningitis before CSF cryptococcal antigen and culture confirmed the fungal etiology [4]. In both instances, as in the present case, the absence of a positive tuberculosis microbiological result and the failure to improve on antituberculosis therapy were critical clues that ultimately guided clinicians toward mycological investigations. In patients with chronic meningitis and negative tuberculosis workup, fungal etiologies must be actively pursued, even in the absence of overt immunodeficiency. Combining India ink staining, cryptococcal antigen testing, and fungal culture early in the diagnostic algorithm can mitigate the “missed opportunity” highlighted in our case and accelerate appropriate antifungal therapy [22–24].

## 4. Conclusion

This case of CM in an immunocompetent patient highlights a critical diagnostic pitfall in tuberculosis‐endemic regions. The combination of subacute headache, CSF hypoglycorrhachia, lymphocytic pleocytosis, and cavitary pulmonary lesions strongly mimics tuberculous meningitis, leading to delayed mycological investigation and initiation of incorrect therapy. The eventual diagnosis through India ink staining and fungal culture underscores the necessity of including these simple, low‐cost tests in the initial diagnostic workup of any patient presenting with chronic meningitis, particularly when microbiological evidence for tuberculosis is lacking or when the clinical response to ATT is absent. The incidental detection of HHV‐6 DNA likely represented an epiphenomenon rather than a clinically significant co‐infection, reinforcing the importance of re‐evaluating the diagnosis when treatment fails. Prompt recognition of cryptococcal infection enabled effective antifungal therapy, resulting in full clinical recovery and normalization of CSF parameters. Clinicians should maintain a high index of suspicion for fungal meningitis in apparently immunocompetent individuals with chronic meningoencephalitis, as early diagnosis and appropriate treatment remain the cornerstones of favorable outcomes.

## Author Contributions

Ariba Sultan: conceptualization, clinical management, data collection, and drafting of the initial manuscript.

Huda Raja and Nahid Raufi: literature review, preparation of figures and tables, and manuscript editing.

Abdullah Nadeem: conceptualization, supervision, critical revision, and final approval of the manuscript.

Amna Abrar: data acquisition, radiological analysis, and drafting of imaging descriptions.

Shameem Behram Khan: infectious disease consultation, treatment guidance, manuscript review, and overall supervision.

Nahid Raufi (corresponding author): conceptualization, supervision, critical revision, and final approval of the manuscript.

## Funding

The authors declare that they did not receive any specific grant from funding agencies in the public, commercial, or not‐for‐profit sectors for this study.

## Ethics Statement

This case report was conducted in accordance with the ethical standards of the Declaration of Helsinki. Formal ethical approval was waived as per institutional policy for single case reports.

## Consent

Written informed consent was obtained from the patient for participation in this study.

Written informed consent was obtained from the patient for the publication of anonymized clinical data, radiological images, and other relevant findings.

## Conflicts of Interest

The authors declare no conflicts of interest.

## Supporting Information

Additional supporting information can be found online in the Supporting Information section.

## Supporting information


**Supporting Information** This case report has been prepared in accordance with the CAse REport (CARE) guidelines, as outlined in Supporting File 2. Supporting Table 1: Clinical Timeline from Symptom Onset to Follow‐up.

## Data Availability

Data sharing is not applicable to this article as no datasets were generated or analyzed during the current study.
