# Supplementary material for: Cryptococcal Meningitis Beyond Immunosuppression: A Case Report in an Immunocompetent Individual
Source: Case Rep Infect Dis. 2026 Jul 20;2026:8630525. doi: 10.1155/crdi/8630525 (PMC13383004; doi:10.1155/crdi/8630525)
Supplement: Supplementary file 1 — Supporting Information This case report has been prepared in accordance with the CAse REport (CARE) guidelines, as outlined in Supporting File 2. Supporting Table 1: Clinical Timeline from Symptom Onset to Follow‐up. [file CRDI-2026-8630525-s001.zip › CARE-checklist-English-2013-cryto_2.docx]

**CARE Checklist of information to include when writing a case report**

| **Topic** |  | **Item** | **Checklist item description** |  |  | **Reported on Line** |
| --- | --- | --- | --- | --- | --- | --- |

**Title 1** The diagnosis or intervention of primary focus followed by the words “case report” . . . . . . . . . . . . . . . . . . . . 1-2

| **Clinical Findings**  **Timeline**  **Diagnostic**  **Assessment**  **Therapeutic Intervention**  **Follow-up and Outcomes**  **Discussion**  **Patient Perspective**  **Informed Consent** |
| --- |

**Key Words 2** 2 to 5 key words that identify diagnoses or interventions in this case report, including "case report" . . . 32-35

**Abstract 3a** Introduction: What is unique about this case and what does it add to the scientific literature? . . . . . . . . 5-9

**(no references) 3b** Main symptoms and/or important clinical findings . . . . . . . . . . . . . . . . . . . . . . . . . . . . . . . . . . . . . . . . . . . 10-14. . . . . . . . . . . .

**3c** The main diagnoses, therapeutic interventions, and outcomes . . . . . . . . . . . . . . . . . . . . . . . . . . . . . . . . . .18-20 . . . . . . . .

**3d** Conclusion—What is the main “take-away” lesson(s) from this case? . . . . . . . . . . . . . . . . . . . . . . . . . . . . .29-31. . . . . . . .

**Introduction 4** One or two paragraphs summarizing why this case is unique (**may include** reference**s**) . . . . . . . . . . . .53-56

**Patient Information 5a** De-identified patient specific information. . . . . . . . . . . . . . . . . . . . . . . . . . . . . . . . . . . . . . . . . . . . . . . . . 58-60. . .

**5b** Primary concerns and symptoms of the patient. . . . . . . . . . . . . . . . . . . . . . . . . . . . . . . . . . . . . . . . . . . . 60-66 . . . . . . . .

**5c** Medical, family, and psycho-social history including relevant genetic information . . . . . . . . . . . . . . . . 58-66.

**5d** Relevant past interventions with outcomes . . . . . . . . . . . . . . . . . . . . . . . . . . . . . . . . . . . . . . . . . . . . . . .84-117 . . . . .

1. Describe significant physical examination (PE) and important clinical findings. . . . . . . . . . . . . . . . . . 67-75 .
2.
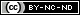

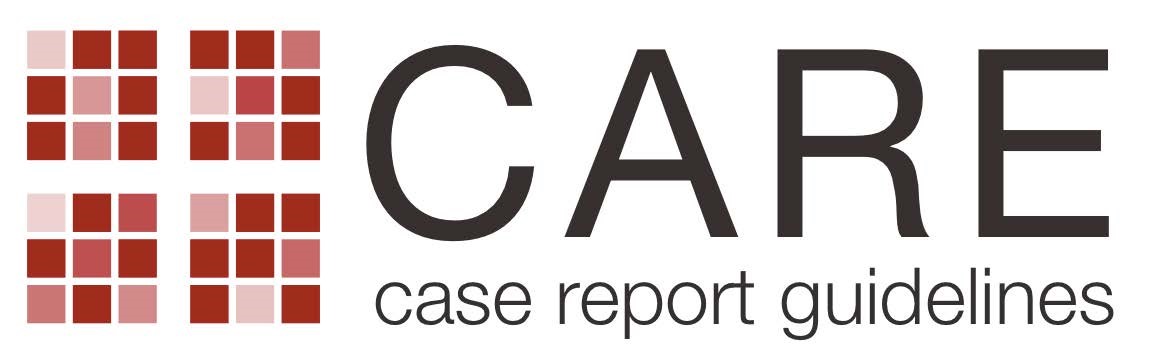
Historical and current information from this episode of care organized as a timeline . . . . . . . . . . . . . . 60-75.

**8a** Diagnostic testing (such as PE, laboratory testing, imaging, surveys). . . . . . . . . . . . . . . . . . . . . . . . . . . . . . .76

**8b** Diagnostic challenges (such as access to testing, financial, or cultural) . . . . . . . . . . . . . . . . . . . . . . . . . . . . 84.

**8c** Diagnosis (including other diagnoses considered) . . . . . . . . . . . . . . . . . . . . . . . . . . . . . . . . . . . . . . . . . . . . . . .84 . .

**8d** Prognosis (such as staging in oncology) where applicable . . . . . . . . . . . . . . . . . . . . . . . . . . . . . . . . . . . . . . . . . na

**9a** Types of therapeutic intervention (such as pharmacologic, surgical, preventive, self-care) . . . . . . . . . . . . . . . 96. . . . .

**9b** Administration of therapeutic intervention (such as dosage, strength, duration) . . . . . . . . . . . . . . . . . . . . . . . 96. . . . . .

**9c** Changes in therapeutic intervention (with rationale) . . . . . . . . . . . . . . . . . . . . . . . . . . . . . . . .. . . . . . . . 97-117. . . . . . .

**10a** Clinician and patient-assessed outcomes (if available) . . . . . . . . . . . . . . . . . . . . . . .. . . . . . . . . . . . . . . 113-117. . . . . . . . . . . . .

**10b** Important follow-up diagnostic and other test results . . . . . . . . . . . . . . . . . . . . . . . . . . . . . . . . . . . . . . 113-117. . . . . . . . . . . . . .

**10c** Intervention adherence and tolerability (How was this assessed?) . . . . . . . . . . . . . . . . . . . . . . . .97-117 . . . . . . . . . . . . . . . **10d** Adverse and unanticipated events . . . . . . . . . . . . . . . . . . . . . . . . . . . . . .97-117 . . . . . . . . . . . . . . . . . . . . . . . . . . . . . . . . . . . . .

**11a** A scientific discussion of the strengths AND limitations associated with this case report . . . . . . 165-180. . . .

**11b** Discussion of the relevant medical literature **with references**. . . . . . . . . . . . . . . . . . . . . . . . . . . . 165-170. . .. . .

**11c** The scientific rationale for any conclusions (including assessment of possible causes) . . . . . . . . . . .177-180 . . .

**11d** The primary “take-away” lessons of this case report (without references) in a one paragraph conclusion . . . .179-180 . . .

1. The patient should share their perspective in one to two paragraphs on the treatment(s) they received . . . . . . . . na
2. Did the patient give informed consent? Please provide if requested . . . . . . . . . . . . . . . . . . . . . . . . . . . . . . . . . . . . . .  **Yes**
