# Supplementary material for: Cryptococcal Meningitis Beyond Immunosuppression: A Case Report in an Immunocompetent Individual
Source: Case Rep Infect Dis. 2026 Jul 20;2026:8630525. doi: 10.1155/crdi/8630525 (PMC13383004; doi:10.1155/crdi/8630525)
Supplement: Supplementary file 1 — Supporting Information This case report has been prepared in accordance with the CAse REport (CARE) guidelines, as outlined in Supporting File 2. Supporting Table 1: Clinical Timeline from Symptom Onset to Follow‐up. [file CRDI-2026-8630525-s001.zip › supp table.docx]

**Supplementary Table 1: Clinical Timeline from Symptom Onset to Follow‑up**

| **Timepoint** | **Key Events** |
| --- | --- |
| **2 months prior to admission** | Gradual onset of diffuse, dull‑aching headache; small‑quantity vomiting (self‑resolved). |
| **15 days prior to admission** | Bilateral lower limb pain followed by subjective weakness (no objective motor deficit). |
| **Day 0 – Admission** | Emergency department presentation. Normal vital signs, GCS 15, no meningeal signs. Initial CSF: glucose 1 mg/dL, protein 356 mg/dL, WBC 140/mm³ (70% lymphocytes). Gram stain, AFB smear, GeneXpert, and bacterial cultures negative. MRI brain: leptomeningeal enhancement. |
| **Day 0–2** | Presumptive tuberculous meningitis. Empirical anti‑tuberculous therapy (ATT) started. Chest imaging: bilateral fibrotic changes, cavitary nodule (RUL 1.2×1.1 cm), calcified lymph nodes. |
| **Day ~3** | Beta‑D‑glucan elevated (80.41). Voriconazole started; BAL samples sent. Repeat CSF: glucose 13 mg/dL, protein 191 mg/dL, WBC 80/mm³. CSF HHV‑6 PCR positive → intravenous valacyclovir initiated. |
| **Day ~5 (approx.)** | No clinical improvement with antivirals. Voriconazole discontinued. Empirical intravenous liposomal amphotericin B + fluconazole started. CSF re‑sent for India ink and fungal culture. |
| **Day ~7–10** | India ink preparation positive; CSF culture grows *Cryptococcus neoformans*. Diagnosis confirmed. ATT and valacyclovir stopped. HIV and HbA1c negative. |
| **After 2 weeks of amphotericin B/fluconazole (approx. Day 21)** | Repeat CSF: clear, glucose 74 mg/dL, protein 93 mg/dL, WBC 65/mm³. Fungal culture no growth. Headache resolved; motor symptoms absent. |
| **Discharge (approx. Day 21–24)** | Consolidation phase: oral fluconazole 400 mg/day for 8 weeks. |
| **Week 12** | Maintenance phase: fluconazole 200 mg/day for 6 months. |
| **Follow‑up** | Sustained clinical improvement. Renal function and electrolytes normal. No neurological sequelae. |
